# Ubiquitination of CD47 Regulates Innate Anti‐Tumor Immune Response

**DOI:** 10.1002/advs.202412205

**Published:** 2024-12-12

**Authors:** Qian Gou, Bingjun Yan, Yalan Duan, Yilei Guo, Jing Qian, Juanjuan Shi, Yongzhong Hou

**Affiliations:** ^1^ School of Life Science Jiangsu University Zhenjiang Jiangsu Province 212013 People's Republic of China

**Keywords:** autophagy, CD47, immune escape, TRAF2, ubiquitination

## Abstract

In addition to adaptive immune checkpoint of PD‐1/PD‐L1, the innate immune checkpoint SIRPα/CD47 plays an important role in regulation of tumor immune escape. However, the mechanism of CD47 ubiquitination on tumor immune escape remains unclear. Here it is found that TRAF2 bound to the C‐terminal of CD47 cytoplasmic fragment and induced its ubiquitination, leading to inhibition of CD47 autophagic degradation by disrupting its binding to LC3, which in turn inhibited macrophage phagocytosis and promoted tumor immune escape. In contrast, loss of TRAF2 facilitated CD47 autophagic degradation and inhibited tumor immune escape. Moreover, autophagy induction promoted CD47 degradation and enhanced the efficacy of CD47 antibody anti‐tumor immunotherapy. These findings revealed a novel mechanism of ubiquitination of CD47 on tumor immune escape.

## Graphical abstract

TRAF2 induced CD47 ubiquitination leading to inhibition of CD47 autophagic degradation, which in turn blocked macrophage phagocytosis. In contrast, the loss of TRAF2 reversed this event. Moreover, autophagy induction facilitated CD47 autophagic degradation and enhanced CD47 antibody anti‐tumor immunotherapy.

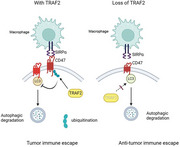

## 1 Introduction

The innate immune system of host serves as an essential role in inhibiting tumor progression by killing cancer cells,^[^ ^1^, ^2^ ^]^ while cancer cell could escape immune surveillance by expressing high levels of CD47 (cluster of differentiation 47).^[^ ^3^ ^]^ As innate immune response, the SIRPα (signal‐regulatory protein α)/CD47 immune checkpoint acts as a “don't eat me” signal.^[^ ^4^, ^5^ ^]^ The immune checkpoint of SIRPα/CD47 was first identified in 1999.^[^ ^6^, ^7^ ^]^ CD47 on cancer cells binds to SIRPα on phagocytes, leading to inhibition of its phagocytosis of cancer cells,^[^ ^4^, ^5^, ^8^ ^]^ and promotes tumor immune escape.^[^ ^3^, ^9^ ^]^ As a transmembrane glycoprotein, high expression of CD47 protein is detected on multiple types of cancer including colon, bladder, ovarian, glioblastoma, breast, and hepatocellular carcinoma.^[^ ^10^ ^]^ CD47 gene expression is regulated by c‐Myc, NFκB, and HIF contributing to tumor immune escape.^[^ ^2^, ^8^, ^9^, ^11^, ^12^ ^]^ However, the effect of ubiquitination of CD47 on tumor immune evasion remains unclear. Protein ubiquitination modification contains three steps enzymatic process, which includes E1 ubiquitin‐activating enzyme, E2 ubiquitin‐conjugating enzyme, and E3 ubiquitin ligase.^[^ ^13^, ^14^ ^]^ Among them, E3 ubiquitin ligase plays an important role in regulating protein ubiquitination.^[^ ^15^ ^]^ As one of the E3 ubiquitin ligases, TRAF2 can induce protein ubiquitination, which in turn regulates NFκB pathway‐mediated pro‐inflammatory response or tumor progression.^[^ ^16^, ^17^, ^18^, ^19^, ^20^ ^]^ UCHL3 could increase TRAF2 protein stability and activate NFκB‐mediated ovarian cancer progression.^[^ ^16^ ^]^ In addition, cIAP1/cIAP2 facilitates TRAF2‐mediated IKKε ubiquitination leading to NFκB‐induced breast cancer development.^[^ ^17^ ^]^ TRAF2‐RIP‐TAK1‐IKK pathway activates NFκB and promotes head and neck cancer cell proliferation.^[^ ^18^ ^]^ In colon cancer, high expression of TRAF2 activates NFκB pathway resulting in tumor progression.^[^ ^19^ ^]^ In addition to activation of NFκB pathway, TRAF2 can induce RSK2 ubiquitination, leading to an increase in RSK2 activity, subsequently, facilitates AP‐1‐mediated tumor progression,^[^ ^21^ ^]^ and TRAF2‐mediated ubiquitination of p62 results in activation of mTORC1 pathway, which in turn promotes proliferation of liver cancer cells.^[^ ^22^ ^]^ However, the interaction of TRAF2 with CD47 on tumor immune escape is still unclear. Here we found that TRAF2 induced CD47 ubiquitination, leading to inhibition of CD47 autophagic degradation, which in turn facilitated tumor immune escape.

## 2 Results

### 2.1 Correlation of TRAF2 with CD47 and Macrophage

**Figure 1:**
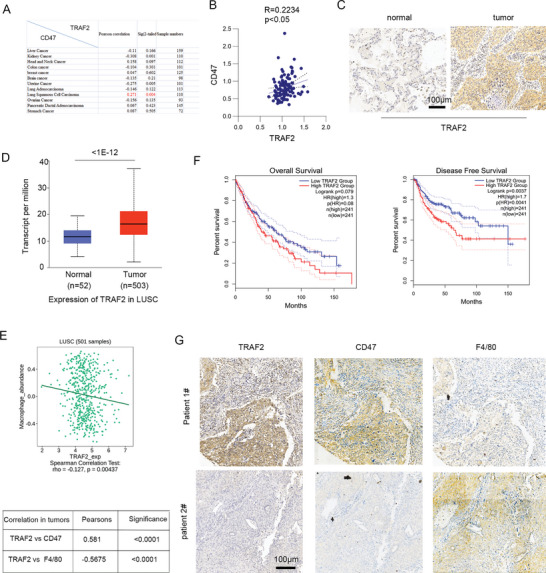
The clinical correlation of TRAF2 with CD47 and macrophages. A) The protein expression correlation of TRAF2 with CD47 in different types of cancer was analyzed using The Cancer Proteogenomic Data Analysis Site (cProSite). B) The significantly positive correlation of TRAF2 expression levels with CD47 in lung squamous cell carcinoma (LUSC), *n* = 110. C) Immunohistochemistry staining of TRAF2 in normal or tumor tissues of LUSC. Scale bar: 100 µm. D) The gene expression levels of TRAF2 in normal or tumor tissues of LUSC derived from TCGA database. E) The correlation of TRAF2 expression with macrophage abundance was assayed using TISIDB (hku.hk). F) The survival of TRAF2 expression in LUSC derived from GEPIA 2. G) Immunohistochemistry analysis of TRAF2, CD47, and macrophages using LUSC specimens. The correlation of TRAF2 with CD47 and macrophage was assayed (*n* = 48). Scale bar: 100 µm.

The Clinical Proteomic Tumor Analysis Consortium (CPTAC) suggests that the expression of TRAF2 is significantly positive correlated with CD47 protein levels in lung squamous cell carcinoma (LUSC) among 12 types of tumor (**Figure** **1**A,B). Immunohistochemical staining analysis using tumor tissues showed that TRAF2 protein level was higher in LUSC than that of normal tissue (Figure 1C), which was consistent with high TRAF2 gene expression in LUSC (Figure 1D). Since the binding of CD47 on cancer cells to SIRPα on macrophage results in inhibition of macrophage phagocytosis,^[^ ^2^, ^3^ ^]^ bioinformatic analysis using TISIDB database suggests that TRAF2 expression is negatively correlated with macrophage abundance in LUSC (Figure 1E). Consistently, high expression of TRAF2 correlates with poor overall survival and disease‐free survival in patients (Figure 1F). Further analysis showed that high expression of TRAF2 correlated with high expression of CD47 and low levels of macrophage infiltration in LUSC tissues (Figure 1G). These findings suggest that TRAF2 was negatively correlated with macrophage infiltration, which was involved in high expression of CD47.

### 2.2 Loss of TRAF2 Inhibited Tumor Immune Escape

**Figure 2:**
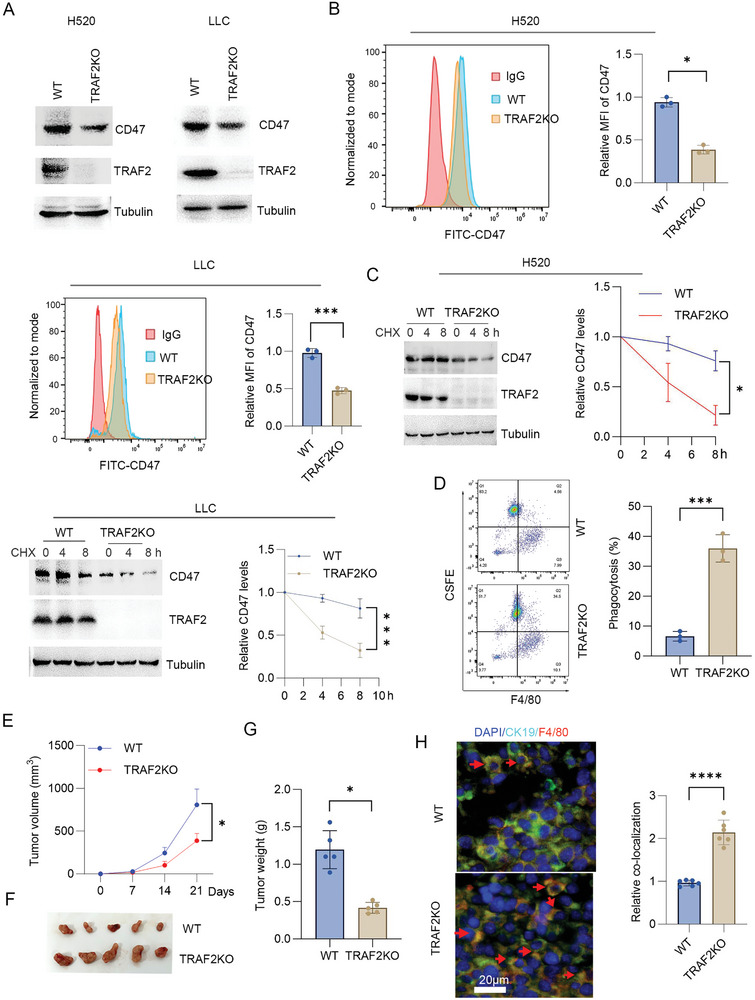
Loss of TRAF2 inhibited tumor immune escape by increasing phagocytosis. A) Western blot analysis of WT or TRAF2^−/−^ H520 or LLC cells. B) Flow cytometry analysis of surface CD47 levels in WT or TRAF2^−/−^ H520 or LLC cells. MFI: median fluorescence intensity. Results are expressed as means ± SD (*n* = 3). C) Half‐life analysis of CD47 protein level in WT or TRAF2^−/−^ H520 or LLC cells. Cells were treated with cycloheximide (30 µg mL^−1^) as indicated time course. The relative CD47 protein level was quantified. Results are expressed as means ± SD (*n* = 3). D) phagocytosis of WT or TRAF2^−/−^ H520 cells. Results are expressed as means ± SD (*n* = 3). E–G) WT or TRAF2^−/−^ LLC cells were inoculated subcutaneously into C57BL mice. Tumor volume and weight were measured. Results are expressed as means ± SD, *n* = 5. H) Immunofluorescence of tumor tissues. Percent co‐localization of macrophage (F4/80) with cancer cells (CK19, a cancer cell marker) was quantified. Results are expressed as means ± SD (*n* = 6 fields). Scale bar: 20 µm. Red arrow: co‐location.

To detect the effect of TRAF2 on CD47 expression, TRAF2 gene knockout cells were developed by CRISPR/Cas9. The results showed that loss of TRAF2 reduced CD47 protein levels in H520, LLC, H1975, and H226 cells (**Figure** **2**A; Figure S1A, Supporting Information). Consistently, loss of TRAF2 reduced the surface CD47 levels by flow cytometry analysis (Figure 2B; Figure S1B, Supporting Information). To detect the effect of TRAF2 on CD47 gene levels, qPCR analysis was performed. The results showed that loss of TRAF2 had no significant effect on CD47 gene levels (Figure S2, Supporting Information), suggesting that TRAF2 increased CD47 protein stability. Half‐life analysis showed that loss of TRAF2 reduced CD47 protein stability in H520 or LLC cells (Figure 2C). These findings suggest that TRAF2 inhibited CD47 protein degradation. To further examine the effect of TRAF2 on macrophage phagocytosis, co‐cultured macrophages with TRAF2 knockout H520 cells was performed. The results showed that TRAF2 knockout increased macrophage phagocytosis (Figure 2D). To detect the effect of TRAF2 on tumor immune escape, TRAF2 gene knockout LLC cells were implanted into C57/BL immunocompetent mice. These results showed that TRAF2 knockout inhibited tumor growth (Figure 2E), and decreased tumor weight (Figure 2F,G). Immunostaining analysis showed that TRAF2 knockout markedly increased the colocalization of tumor cells with macrophages (Figure 2H), suggesting that TRAF2 knockout increased the macrophage phagocytosis. These findings suggest that TRAF2 promoted tumor immune escape by increasing CD47 protein stability and inhibiting phagocytosis.

### 2.3 The Interaction of TRAF2 with CD47

**Figure 3:**
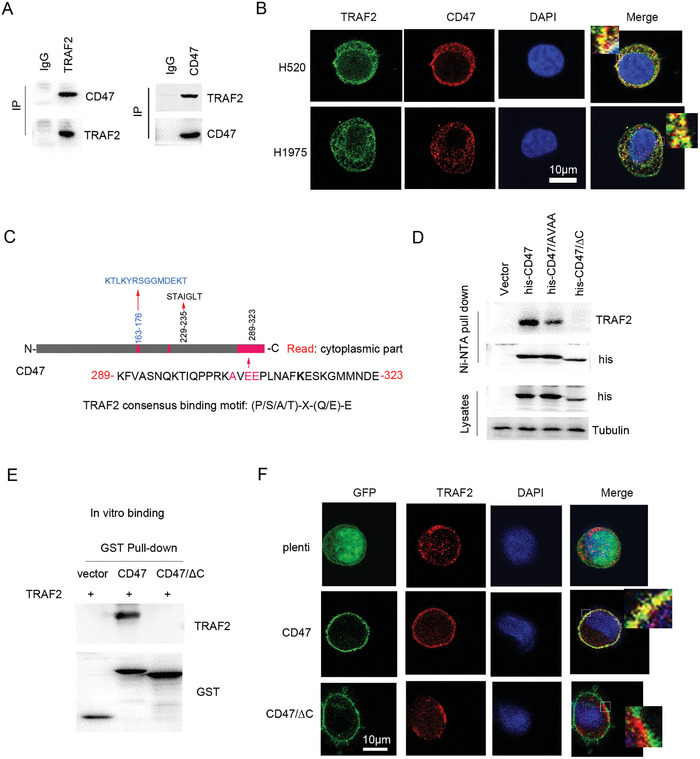
The interaction of TRAF2 with CD47. A) Immunoprecipitation analysis of H520 cell lysates. B) Confocal analysis of cells. Scale bar: 10 µm. C) Schematic illustration of CD47. D) H520 cells were transfected plasmids as indicated. Cell lysates were subjected to Ni‐NTA pull‐down and Western blot. E) In vitro binding assay of TRAF2 with CD47 as described in Experimental Section. F) Confocal analysis of H520 cells expressed different genes as indicated. Scale bar: 10 µm.

Under the basal condition, immunoprecipitation analysis showed that TRAF2 bound to CD47 (**Figure** **3**A; Figure S3, Supporting Information). Confocal analysis further confirmed that TRAF2 co‐localized with CD47 in lung cancer cells (Figure 3B). Previous study shows that TRAF2 can bind the substrates with (P/S/A/T)‐X‐(Q/E)‐E motif, where X refers any amino acid.^[^ ^23^ ^]^ Interestingly, the C‐ terminal of CD47 cytoplasmic fragment contains TRAF2 binding motif (AVEE) (Figure 3C). Ni‐NTA pull‐down analysis showed that the mutant of CD47/AVAA inhibited the binding of TRAF2 to CD47, whereas the mutant of the C‐terminal of CD47 completely blocked this interaction (Figure 3D), suggesting that CD47 C‐terminal was required for TRAF2 binding, which was further demonstrated by in vitro binding analysis (Figure 3E). Additional, confocal analysis showed that deleted C‐terminal of CD47 inhibited the co‐localization of CD47 with TRAF2 (Figure 3F). These findings suggest that TRAF2 bound to the C‐terminal region of CD47.

### 2.4 TRAF2 Induced CD47 Ubiquitination and Increased its Protein Stability

**Figure 4:**
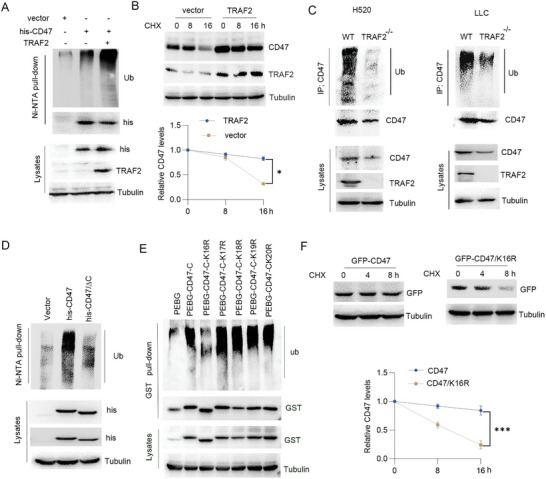
TRAF2‐induced CD47 ubiquitination and inhibited its protein degradation. A) H520 cells were transfected plasmids as indicated for 48 h. Ubiquitination of CD47 was assayed. B) H520 cells were transfected vector or TRAF2 plasmids for 48 h. Cells were treated with cycloheximide (30 µg mL^−1^) as indicated time course. The relative CD47 protein level was quantified. Results are expressed as means ± SD (*n* = 3). C) Immunoprecipitation and Western blot analysis were performed using TRAF2^−/−^ H520 or LLC cell lysates. D) H520 cells were transfected plasmids as indicated for 48 h. Cell lysates were subjected to Ni‐NTA pull‐down and Western blot. E) H520 cells were transfected plasmids as indicated. Cell lysates were subjected to GST pull‐down and Western blot. F) H520 cells were transfected plasmids as indicated. Cells were treated with cycloheximide (30 µg mL^−1^) as indicated time course. The relative CD47 protein level was quantified. Results are expressed as means ± SD (*n* = 3).

As an E3 ubiquitin ligase, TRAF2 could induce the substrates ubiquitination.^[^ ^16^, ^17^, ^18^, ^19^ ^]^ To detect the effect of TRAF2 on CD47 ubiquitination, TRAF2 was overexpressed in cells and the results showed that TRAF2 significantly induced CD47 ubiquitination (**Figure** **4**A), and increased CD47 half‐life (Figure 4B). In contrast, loss of TRAF2 significantly blocked CD47 ubiquitination in H520 or LLC cells (Figure 4C). The above results showed that the CD47 C‐terminal was required for TRAF2 binding, further analysis showed that TRAF2 induced CD47 ubiquitination, but the mutant of CD47/∆C significantly inhibited this event (Figure 4D). To detect the specific ubiquitinated residue, prediction analysis using the online BDM‐PUB software suggested that lysine‐16 of CD47 C‐terminal is the potential ubiquitinated site. GST pull‐down analysis showed that the K16 mutant of CD47 significantly inhibited its ubiquitination (Figure 4E), suggesting that TRAF2 could induce CD47 C‐terminal lysine‐16 residue ubiquitination. Since TRAF2‐mediated CD47 ubiquitination led to increased CD47 protein stability, further analysis showed that the CD47/K16R mutant significantly reduced CD47 half‐life (Figure 4F). These findings suggest that TRAF2 induced ubiquitination at lysine‐16 residue in the CD47 C‐terminal resulting in inhibition of CD47 degradation.

### 2.5 Ubiquitination of CD47 Promoted Tumor Immune Escape by Inhibiting Phagocytosis

**Figure 5:**
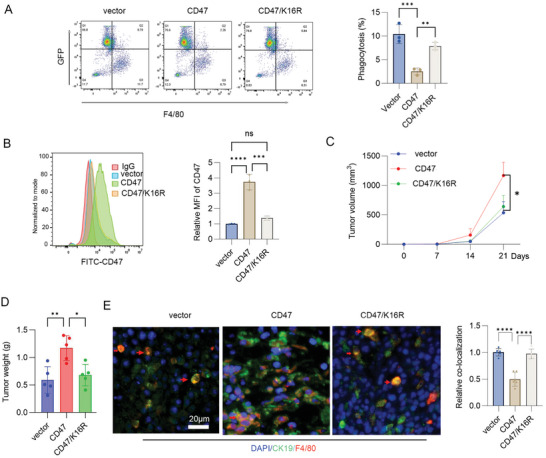
TRAF2/CD47 pathway promoted tumor immune escape by inhibiting phagocytosis. A) phagocytosis analysis of the expression of CD47 or CD47/K16R gene in H520 cells. Results are expressed as means ± SD (*n* = 3). B) Membrane CD47 level was assayed using flow cytometry in CD47 or CD47/K16R stable expression H520 cells. MFI: median fluorescence intensity. Results are expressed as means ± SD (*n* = 3). C,D) CD47 or CD47/K16R stable expression LLC cells were inoculated subcutaneously into C57BL mice. Tumor volume and weight were measured. Results are expressed as means ± SD, *n* = 5. E) Immunofluorescence of tumor tissues. Percent colocalization of macrophage (F4/80) with cancer cells (CK19) was quantified. Results are expressed as means ± SD (*n* = 6 fields). Scale bar: 20 µm. DAPI: blue, CK19: green, F4/80: red.

The above results showed that the CD47/K16R mutant inhibited the ubiquitination of CD47 and reduced CD47 protein stability. Consistently, CD47/K16R mutant significantly reversed the inhibition of CD47 on macrophage phagocytosis compared to overexpression of CD47 (**Figure** **5**A), which was associated with reduced surface CD47 levels in CD47/K16R expressing cells (Figure 5B). As the lysine‐16 of CD47 C‐terminal is highly conserved between human and mice (Figure S4, Supporting Information), mice CD47/K16R mutant was constructed. Implanted tumor model analysis showed that overexpressed CD47 markedly promoted tumor growth and increased tumor weight, but CD47/K16R mutant reversed this event (Figure 5C,D). Immunofluorescence analysis showed that overexpression of CD47 inhibited the co‐localization of cancer cells with macrophages compared to CD47/K16R mutant (Figure 5E), suggesting that overexpression of CD47 inhibited phagocytosis, whereas the CD47/K16R mutant reversed this event. These findings suggest that TRAF2 increased CD47 stability by inducing its ubiquitination, leading to inhibition of phagocytosis and promotion of tumor immune escape.

### 2.6 The Binding of CD47 to LC3

**Figure 6:**
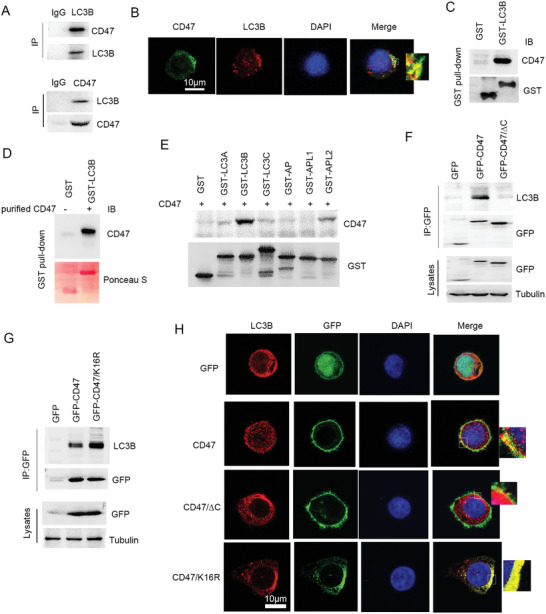
The interaction of CD47 with LC3B. A) Immunoprecipitation of cell lysates. B) Confocal analysis of the interaction of LC3B with CD47. Scale bar: 10 µm. C) H520 cells were transfected with PEBG or PEBG‐LC3B plasmids for 48 h. Cell lysates were subjected to GST pull‐down and Western blot. D) In vitro binding analysis of CD47 with LC3B as described in Experimental Section. E) In vitro binding analysis of CD47 with ATG8 family proteins as described in Experimental Section. F) H520 cells were transfected with GFP‐CD47 or GFP‐CD47/∆C plasmids for 48 h. Cell lysates were subjected to immunoprecipitation and Western blot. G) H520 cells were transfected with GFP‐CD47 or GFP‐CD47/K16R plasmids for 48 h. Cell lysates were subjected to immunoprecipitation and Western blot. H) Stable expression of CD47, CD47/∆C, or CD47/K16R in H520 cells was subjected to confocal analysis. Scale bar: 10 µm.

The above results showed that TRAF2 inhibited CD47 protein degradation, while the mechanism of CD47 degradation is still unclear. To detect whether CD47 could be degradation by autophagy, cancer cells were treated with CQ (lysosome inhibitor). The results showed that CD47 protein levels had accumulated after treatment with CQ (Figure S5, Supporting Information), suggesting that CD47 underwent autophagic degradation under basal conditions. LC3B is the ATG8 family proteins, which plays an important role in autophagosome formation.^[^ ^24^ ^]^ Immunoprecipitation analysis showed that CD47 bound to LC3B in different types of cancer cells (**Figure** **6**A; Figure S6, Supporting Information). Confocal analysis showed that CD47 co‐localized with LC3B (Figure 6B). GST pull‐down analysis showed that CD47 bound to LC3B (Figure 6C), which was further demonstrated by in vitro binding analysis (Figure 6D). The ATG8 family proteins include LC3A, LC3B, LC3C, GABARAP (AP), GABARAPL1 (APL1), and GABARAPL2 (APL2).^[^ ^24^, ^25^ ^]^ In vitro binding assay showed that CD47 bound significantly more to LC3B compared to other ATG8 family proteins (Figure 6E). These findings suggest that CD47 could directly bind to LC3B. Immunoprecipitation analysis showed that deleted C‐terminal of CD47 abolished the binding of CD47 to LC3B (Figure 6F), suggesting that the C‐terminal of CD47 was required for LC3B binding. Since TRAF2 induced CD47 ubiquitination at lysine‐16 residue, further analysis showed that CD47/K16R mutant significantly increased the binding of CD47 to LC3B (Figure 6G). The confocal analysis further demonstrated that the CD47/K16R mutant was significantly bound to LC3B compared to the wild type, whereas the CD47/∆C mutant abolished the binding of LC3B to CD47 (Figure 6H). These findings suggest that TRAF2‐mediated CD47 ubiquitination led to disruption of the binding of LC3B to CD47.

### 2.7 CD47 Underwent Autophagic Degradation

**Figure 7:**
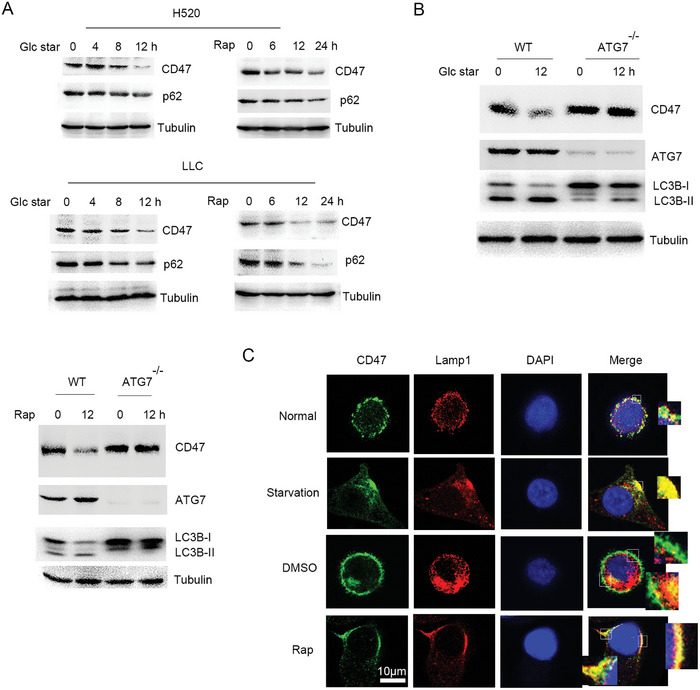
Autophagy induction promoted CD47 autophagic degradation. A) Western blot analysis of H520 or LLC cell lysates in response to glucose starvation or rapamycin (2 µm) as indicated time course. B) Western blot analysis of WT or ATG7^−/−^ H520 cells treated with glucose starvation or rapamycin (2 µm). C) Confocal analysis the co‐localization of CD47 with lamp1 in H520 cells treated with glucose starvation or rapamycin (2 µm) for 4 h. Scale bar: 10 µm.

Autophagy is intracellular materials degradation procession that occurs in response to stimuli such as starvation or rapamycin treatment.^[^ ^26^ ^]^ Western blot analysis showed that CD47 protein was significantly reduced in response to glucose starvation or rapamycin (**Figure** **7**A; Figure S7, Supporting Information). In addition, although autophagy induction significantly reduced CD47 protein levels, blockade of autophagy using ATG7 gene knockout cells resulted in inhibition of CD47 autophagic degradation (Figure 7B). Confocal analysis showed that CD47 markedly co‐localized with lamp1 in response to autophagy induction (Figure 7C). These findings suggest that the binding of CD47 to LC3 promoted CD47 autophagic degradation in response to autophagy induction.

### 2.8 Autophagy Induction Enhanced Anti‐Tumor Immunotherapy by Inhibiting TRAF2/CD47 Pathway

**Figure 8:**
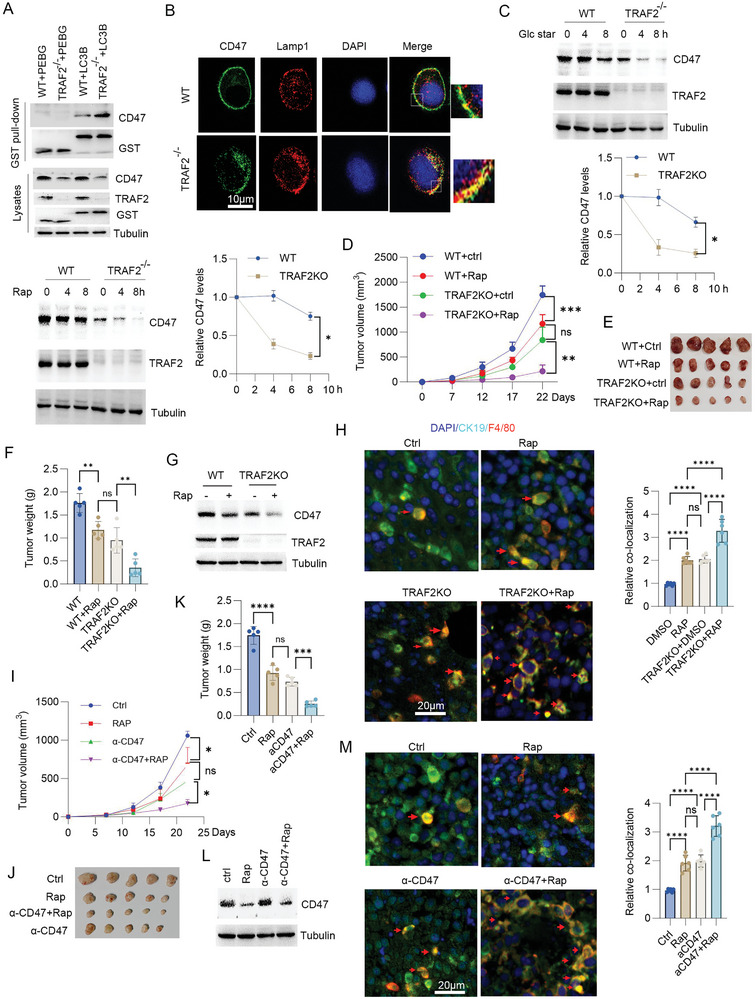
Autophagy induction enhanced CD47 antibody anti‐tumor immunotherapy. A) WT or TRAF2^−/−^ H520 cells were transfected with PEBG or PEBG‐LC3B plasmids. Cell lysates were subjected to GST pull‐down and Western blot. B) Confocal analysis of WT or TRAF2^−/−^ H520 cells. Scale bar: 10 µm. C) Half‐life analysis of WT or TRAF2^−/−^ H520 cells in response to glucose starvation or rapamycin (2 µm). D–F) WT or TRAF2^−/−^ LLC cells were inoculated subcutaneously into C57BL mice. Mice were treated without or with rapamycin as described in Experimental Section. Tumor volume and weight were measured. Results are expressed as means ± SD, *n* = 5. G) Western blot analysis of tumor lysates. H) Immunofluorescence of tumor tissues. Percent colocalization of macrophage (F4/80) with cancer cells (CK19) was quantified. Results are expressed as means ± SD (*n* = 6 fields). Scale bar: 20 µm. DAPI: blue, CK19: green, F4/80: red. I–K) LLC cells were inoculated subcutaneously into C57BL/6 mice. Mice were treated without or with rapamycin, anti‐CD47 mice monoclonal antibody, rapamycin+anti‐CD47 antibody as described in Experimental Section. Tumor volume and weight were detected. Results are expressed as means ± SD, *n* = 5. L) Western blot analysis of tumor lysates. M) Immunofluorescence of tumor tissues. Percent colocalization of macrophage (F4/80) with cancer cells (CK19) was quantified. Results are expressed as means ± SD (*n* = 6 fields). Scale bar: 20 µm. DAPI: blue, CK19: green, F4/80: red.

To further detect the effect of TRAF2/CD47 pathway on CD47 autophagic degradation, immunoprecipitation analysis showed that loss of TRAF2 significantly increased the binding of CD47 to LC3B (**Figure** **8**A). Consistently, confocal analysis showed that loss of TRAF2 significantly increased the lysosomal co‐localization of CD47 (Figure 8B). In response to autophagy induction, loss of TRAF2 significantly reduced CD47 half‐life (Figure 8C). These findings suggest that TRAF2 inhibited CD47 autophagic degradation. Further analysis showed that TRAF2 gene knockout significantly increased phagocytosis in response to rapamycin (Figure S8, Supporting Information). Similarly, the combined CD47 antibody with rapamycin significantly enhanced phagocytosis compared to rapamycin or CD47 antibody treatment alone (Figure S9, Supporting Information).

We next detected the effect of autophagy induction on tumor immune escape in TRAF2 gene knockout LLC cell implanted tumor model. The results showed that rapamycin treatment significantly inhibited tumor growth in TRAF2 gene knockout implanted tumor model (Figure 8D–F). Western blot analysis using tumor lysates showed that TRAF2 knockout significantly reduced CD47 levels in response to rapamycin (Figure 8G). Immunofluorescence analysis showed that rapamycin treatment in TRAF2 knockout group significantly increased the co‐localization of cancer cells with macrophages compared to TRAF2 knockout or rapamycin group (Figure 8H), suggesting that TRAF2 knockout increased phagocytosis in response to rapamycin. Importantly, the combined CD47 antibody with rapamycin significantly enhanced anti‐tumor immunotherapy (Figure 8I–K). Western blot analysis using tumor lysates showed that rapamycin reduced CD47 protein levels (Figure 8L). Additionally, immunofluorescence analysis showed that the combined CD47 antibody with rapamycin significantly increased the co‐localization of cancer cells with macrophages compared to rapamycin or CD47 antibody treatment alone (Figure 8H), suggesting that this combination treatment promoted phagocytosis. These findings suggest that inhibition of the TRAF2/CD47 pathway enhanced anti‐tumor immunotherapy.

## 3 Discussion

In this study, we demonstrated that TRAF2 induced CD47 ubiquitination, which in turn inhibited CD47 autophagic degradation, and a high level of CD47 on cancer cells blocked macrophage phagocytosis leading to the promotion of NSCLC tumor immune escape, which revealed a novel mechanism of tumor immune escape.

The interaction of CD47 on cancer cells with SIRPα on macrophages leads to the inhibition of phagocytosis, subsequently, promotes tumor immune escape.^[^ ^4^, ^5^ ^]^ CD47 is highly expressed in multiple type of cancers,^[^ ^4^, ^10^, ^27^, ^28^ ^]^ which is up‐regulated by multiple pathways such as Myc,^[^ ^8^ ^]^ NFκB (nuclear factor‐ κB),^[^ ^29^ ^]^ HIF‐1 (hypoxia‐inducible factor 1),^[^ ^12^ ^]^ nuclear respiratory factor 1 (NRF‐1),^[^ ^30^ ^]^ and TCF4.^[^ ^31^ ^]^ In addition to regulating CD47 gene expression, CD47 undergoes pyroglutamate modification in response to QPCTL (glutaminyl‐peptide cyclotransferase‐like).^[^ ^32^ ^]^ Certain amino acids undergo specific modification such as phosphorylation, ubiquitination, glycosylation, s‐nitrosylation, methylation, n‐acetylation, and lipidation, which in turn regulates protein function and stability.^[^ ^33^ ^]^ QPCTL mediates CD47 pyroglutamate formation leading to inhibition of phagocytosis,^[^ ^32^ ^]^ suggesting that CD47 post‐translational modification alters its function. However, the mechanism of CD47 ubiquitination on macrophage phagocytosis remains unclear. Our findings suggest that TRAF2‐medited CD47 ubiquitination led to inhibition of macrophage phagocytosis. In addition, CD47 can be phosphorylated by c‐src kinase.^[^ ^34^ ^]^ In this study, the authors suggest that activation of EGFR promotes the binding of c‐SRC to CD47, leading to c‐Src‐induced CD47 phosphorylation at the Y288 amino acid residue, which subsequently, inhibits TRIM21 E3 ubiquitin ligase‐mediated CD47 degradation. Whereas, since CD47 is a transmembrane protein, and the Y288 amino residue is not in the cytoplasmic fragment. So the mechanism of c‐SRC‐mediated CD47 phosphorylation is still unclear in this report.^[^ ^34^ ^]^ As a transmembrane protein, CD47 contains three cytoplasmic fragments, our findings identified that TRAF2 could directly bind to CD47 in the C‐terminal fragment. Although the C‐terminal of CD47 contains TRAF2 binding motif (P/S/A/T)‐X‐(Q/E)‐E,^[^ ^23^ ^]^ our results showed that this motif was not required for TRAF2 binding, and further demonstrated that the C‐terminal of CD47 was essential for TRAF2 binding. As an E3 ligase, TRAF2 could induce several substrates ubiquitination, leading to activation of NFκB, subsequently, promotes inflammatory response or tumor progression.^[^ ^16^, ^17^, ^18^, ^19^ ^]^ However, the mechanism of TRAF2 on tumor immune evasion is still not clear. Here we identified that TRAF2 is associated with NSCLC immune escape by maintaining CD47 protein stability, leading to inhibition of macrophage phagocytosis.

Protein modification regulates its stability,^[^ ^33^ ^]^ and ubiquitin–proteasome system (UPS) and autophagy pathways both able to induce targeted proteins degradation.^[^ ^35^ ^]^ Other report shows that lysosome inhibitor treatment lead to accumulation of CD47 protein levels in A549 lung cancer cells,^[^ ^36^ ^]^ suggesting that CD47 undergoes autophagic degradation on physiological conditions. In agreement with this, blockade of autophagy using CQ or ATG7 gene knockout lung cancer cells led to increased CD47 protein levels. Autophagy is a catalytic reaction by degrading intracellular components such as proteins or organelles in response to stimuli such as nutrient starvation and hypoxia.^[^ ^35^, ^37^, ^38^ ^]^ In the autophagy process, LC3B, one of ATG8 family members, plays a critical role in selective autophagy.^[^ ^26^ ^]^ Further analysis showed that CD47 bound to LC3B by in vitro and in vivo binding analysis. Interestingly, in response to autophagy induction, CD47 was significant degraded through autophagy. Our findings indicated that CD47 protein autophagic degradation process could be blocked. Since TRAF2 inhibited CD47 degradation, loss of CD47 significantly increased its autophagic degradation in lysosomes. Our findings revealed a novel mechanism of TRAF2‐mediated inhibition of CD47 autophagic degradation.

Whereas SIRPα‐CD47 checkpoint blockade promotes phagocytosis by phagocytes such as macrophages and dendritic cells (DCs) leading to tumor regression by activation of innate immune response.^[^ ^39^, ^40^, ^41^ ^]^ Consistently, induction of autophagy significantly induced CD47 autophagic degradation and promoted macrophage phagocytosis, which in turn inhibited tumor immune escape. Furthermore, induction of autophagy significantly enhanced CD47 antibody anti‐tumor immunotherapy.

**Figure 9:**
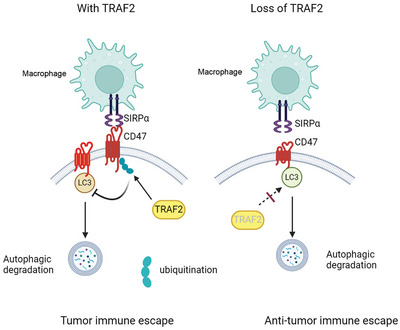
Pathway of TRAF2/CD47 promoted tumor immune escape. TRAF2 induced CD47 ubiquitination leading to inhibition of CD47 autophagic degradation, which in turn blocked macrophage phagocytosis. In contrast, loss of TRAF2 reversed this event. Moreover, autophagy induction facilitated CD47 autophagic degradation and enhanced CD47 antibody anti‐tumor immunotherapy.

In conclusion, TRAF2 induced CD47 ubiquitination, leading to inhibition of CD47 autophagic degradation and increased CD47 protein stability. In contrast, loss of TRAF2 or autophagy induction promoted CD47 autophagic degradation and enhanced CD47 antibody anti‐tumor immunotherapy. Therefore, TRAF2/CD47 pathway regulated tumor innate immune response (**Figure** **9**).

## 4 Experimental Section

### Cells and Reagents

H520, H1975, H226, HEK293T, and LLC cells were purchased from the National Collection of Authenticated Cell Cultures of China. LLC cells were cultured in RPMI 1640 medium supplemented with 10% fetal bovine serum (FBS, Hyclone), while the other cell lines were cultured in DMEM medium supplemented with 10% FBS. A protease inhibitor cocktail was obtained from Sigma. G418 sulfate, cycloheximide(CHX), chloroquine (CQ), rapamycin, and puromycin were purchased from CSN Pharm (China). TRAF2 shRNA plasmid was purchased from GeneCHEM (Shanghai, China).

### Plasmids

The plasmids PGEX‐6P‐LC3A, PGEX‐6P‐LC3B, PGEX‐6P‐LC3C, PGEX‐6P‐Gabarap (AP) were described previously.^[^ ^38^ ^]^ In this study, the following plasmids were generated: PGEX‐6P‐Gabarapl1 (APL1), PGEX‐6P‐Gabarapl2 (APL2), pET‐28a‐CD47, pET‐28a‐CD47/∆C, pcDNA3‐Flag‐TRAF2, pcDNA3‐his‐CD47, pcDNA3‐his/CD47/EE/AA, pcDNA3‐his/CD47/∆C, PEBG‐LC3B, PEBG‐CD47‐C terminal, PEBG‐CD47‐C/K1R, PEBG‐CD47‐C/K2R, PEBG‐CD47‐C/K3R, PEBG‐CD47‐C/K4R, PEBG‐CD47‐C/K5R, pLenti‐GFP‐CD47(mice), and pLenti‐CD47/K1R(mice), pLenti‐GFP‐CD47, pLenti‐GFP‐CD47/∆C, pLenti‐GFP/CD47/K1R were generated in this study.

### Antibodies, Immunoprecipitation, and Western Blot

TRAF2, CD47, and LC3B antibodies were purchased from ImmunoWay Biotechnology. Tubulin, lamp1, ATG7, GST, his, GFP, ubiquitin, and Flag antibodies were purchased from Proteintech. Cells were lysed in lysis buffer (50 mm Tris‐HCl pH7.4, 250 mm NaCl, 0.5% Triton X100, 10% glycerol, 1 mm DTT) supplemented with a protease inhibitor cocktail. The lysates were sonicated and the supernatants were subjected to SDS‐PAGE. After transferring proteins to nitrocellulose membranes, membranes were blocked in TBS‐T with 5% non‐fat milk for 1 h at room temperature. Primary antibodies were diluted in TBST and incubated with membranes overnight at 4 °C. After that, membranes were washed three times with TBST, and then incubated with HRP‐conjugated secondary antibodies (Jackson Immunoresearch) at room temperature for 1h.

For immunoprecipitation, cell lysates were incubated with the primary antibodies for overnight at 4 °C. After that, 12 µL protein A/G magnetic beads (Cat: B23202, Bimake) were added for another 4 h at 4 °C. The beads were washed three times, and the recovered immunocomplexes were resolved by SDS‐PAGE, followed by Western blot analysis with indicated antibodies. Images were developed by chemiluminescence. Blots were quantified by Image J.

### Immunofluorescent Analysis

Cells were washed with PBS and fixed with 3.7% paraformaldehyde for 20 min. After that, cells were washed three times with PBS, and permeabilized with 0.5% Triton‐X100 for 10 min and washed three times. Cells were blocked in 2% BSA/PBS for 1 h, and then incubated with primary antibodies as indicated, washed with PBS for three times, incubated with secondary antibodies (Jackson Immunoresearch). Paraffin‐embedded tumor tissue sections were deparaffinized, rehydrated, and underwent antigen retrieval, respectively.^[^ ^24^ ^]^ Sections were stained with primary antibodies as indicated overnight at 4 °C. After that, sections were washed and incubated with secondary antibodies. Stained cells or tissues were viewed by immunofluorescence microscope.

### In Vitro Binding Analysis

PGEX‐6P‐LC3A, PGEX‐6P‐LC3B, PGEX‐6P‐LC3C, PGEX‐6P‐AP, PGEX‐6P‐APL1, PGEX‐6P‐APL2, PGEX‐6P‐TRAF2, PGEX‐6P‐TRAF2/C34A, pET28a‐CD47, pET28a‐CD47/∆C and PGEX‐TRAF2 were expressed in *E. coli* strain BL21(DE3) [pAPlacIQ]. Protein was purified by Ni‐NTA beads or glutathione beads. For in vitro binding of CD47 to ATG8 family proteins, 5 µg of GST‐LC3A, GST‐LC3B, GST‐LC3C, GST‐AP, GST‐APL1, and GST‐APL2 fusion protein was immobilized on glutathione‐agarose beads in buffer (25 mm HEPES pH7.5, 6 mm NaCl, 0.2% NP‐40) for 1 h at 4 °C, and then the same amount of his‐CD47 protein was added. For in vitro binding of CD47 to TRAF2, 5 µg of GST‐TRAF2 fusion protein was immobilized on glutathione‐agarose beads in buffer (25 mm HEPES pH7.5, 6 mm NaCl, 0.2% NP‐40) for 1 h at 4 °C, and then the same amount of his‐CD47 or his‐CD47/∆C protein was added. These reactions were incubated for another 1h. After that, beads were washed with PBS for three times, and the bound proteins were subjected to Western blot analysis.

### Immunohistochemical Staining

Paraffin‐embedded lung squamous cell carcinoma (LUSC) tumor microarray was obtained from Shanghai Wellbio Technology (China). Tissue sections were deparaffinized, rehydrated, and subjected to antigen retrieval, respectively, as described previously.^[^ ^24^ ^]^ Sections were stained with primary antibodies as indicated overnight at 4 °C. After that, sections were washed and incubated with secondary antibodies at RT for 1 h. Sections were stained with DAB (3,3′‐diaminobenzidine) and counterstained with hematoxylin. This study was reviewed and approved by the Ethics Committee of the Jiangsu University.

### Quantitative Real‐Time PCR

Total RNA was extracted using cell lysates, and CD47 mRNA levels were detected by a Real‐Time PCR assay kit (Takara). Relative mRNA levels were normalized against β‐actin. Fold change over control was determined according to the ΔCt method. CD47 primers: Forward: 5‐GTTGGAGCCATTCTTTTCGTCCC‐3, reverse: 5′‐ATACACGCCGCAATACAGAGACT‐3′; Mice CD47 primers: Forward: 5‐CATTCTGCTGCTCGTTGCCG‐3, reverse: 5′‐ CAGCCCGACCAAAGCAAGGA ‐3′; Human β‐actin primers: Forward: 5′‐GGTGGGCATGGGTCAGAAGGAT‐3′, reverse: 5′‐CACACGCAGCTCATTGTAGAAGGT‐3′. Mice β‐actin primers: Forward: 5′‐CTGACCGAGCG TGGCTACAG ‐3′, reverse: 5′‐ CAGTGGCCATCTCCTGCTCG ‐3′.

### Flow Cytometry

Cells were washed and collected, and then stained with PE anti‐human CD47 (BD biosciences, 568316, 1:250), PE mouse IgG (BD biosciences, 565381, 1:250). PE anti‐human F4/F80 (Elabscinence). After that, cells were washed three times with PBS and analyzed by flow cytometry (CytoFLEX).

### Phagocytosis Assays

Whole blood was obtained from healthy volunteers, and human peripheral blood mononuclear cells (PBMC) were isolated using Ficoll‐Hypaque (GE Healthcare) and selected using a CD14^+^ selection kit (Miltenyi, Biotech). Monocytes were incubated in RMPI 1640 medium supplemented with 10% FBS, 2 mm L‐glutamine, and treated with 50 ng mL^−1^ M‐CSF for 7 days to allow differentiation into macrophages. For the phagocytosis assay, cancer cells were labeled with carboxyfluorescein succinimidyl ester (CFSE) (Beyotime). Macrophages were co‐cultured with cancers at 1:4 ratio in serum‐free medium for 4 h. Collected cells were stained with anti‐F4/80 antibody (eBioscience) and detected using flow cytometry. The phagocytosis rate was calculated as the percentage of F4/80^+^CFSE^+^ or F4/F80^+^GFP^+^ cells relative to the total number of total F4/80^+^ cells.

### Gene Knockout Cell Lines

ATG7 gene knockout in H520 cells was described previously.^[^ ^42^ ^]^ Human or mice TRAF2 gene knockout cells were developed using CRISPR/Cas9 method. The constructs pYSY‐CMV‐Cas9‐U6‐TRAF2(human)‐ sgRNA1‐EFla‐neo, and pYSY‐CMV‐Cas9‐U6‐TRF2(mice)‐sgRNA1‐EFla‐neo were provided by YST BioTech (China). TRAF2 (human) sgRNA: GCAGCTAGCGTGACCCCCCC; TRAF2 (mice) sgRNA: GCTTCTCCAAGACCCTCCTG; These plasmids were transfected into HEK293T cells to produce lentiviral particles, which were then used to infect H520 or LLC cells. Infected cells were selected with G418.

### Implanted Mice Tumor Model

WT or TRAF2^−/−^ LLC cells (1 × 10^5^) were injected subcutaneously into 5‐week‐old C57BL/6 male mice. The pLenti‐CD47 and pLenti‐CD47/K1R plasmids were transfected into 293T cells to produce lentiviral particles, and collected lentiviral particles were infected LLC cells. Stably expressing CD47 or CD47/K1R LLC cells (1 × 10^5^) were injected subcutaneously into 5‐week‐old C57BL/6 male mice. In other studies, C57BL/6 mice were treated with Rapamycin (0.5mg kg^−1^ day^−1^), intratumoral injection of CD47 antibody (100 µg per mice, BioXcel), or Rapamycin (0.5 mg kg^−1^ day^−1^) +CD47 antibody (100 µg per mice, BioXcel). Tumor volume was measured with a digital caliper. Tumor volume = 1/2 (length × width^2^). All mice were obtained from the Animal Center of Jiangsu University. All studies were carried out with the approval of the Jiangsu University Animal Care Committee.

### Statistical Analysis

Data are expressed as the mean ± SD, and all statistical analyses were performed using GraphPad Prism 10.1.2 software. Differences between two dependent groups were evaluated with *t*‐test. One‐way ANOVA or two‐way ANOVA was used to compare more than two groups. A *p*‐value of *p* < 0.05 was accepted as being statistically significant, with significance levels indicated as follows: *p < 0.05, **p < 0001, ***p < 0.001.

## Conflict of Interest

The authors declare no conflict of interest.

## Supporting information



Supporting Information

## Data Availability

The data that support the findings of this study are available from the corresponding author upon reasonable request.
