# Supplementary material for: Ubiquitination of CD47 Regulates Innate Anti‐Tumor Immune Response
Source: Adv Sci (Weinh). 2024 Dec 12;12(5):2412205. doi: 10.1002/advs.202412205 (PMC11792004; doi:10.1002/advs.202412205)
Supplement: Supplementary file 1 — Supporting Information [file ADVS-12-2412205-s001.docx]

**Supplemental Figures and legends**

**
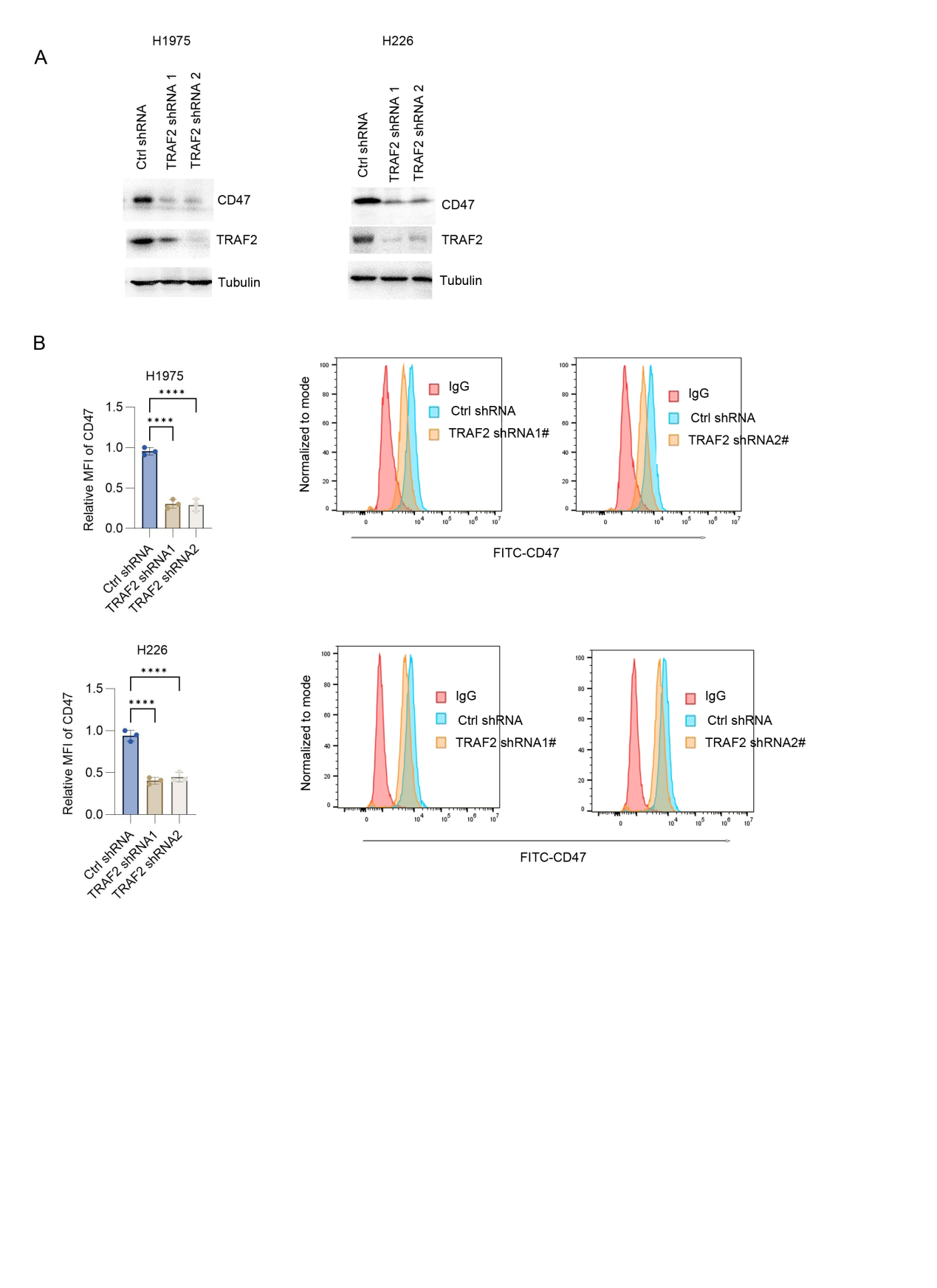
**

**Figure S1. TRAF2 reduced CD47 protein levels**

**A,** Western blot analysis of lysates derived from TRAF2 silenced cells. **B,** Flow cytometry analysis of surface CD47 levels in TRAF2 silenced cells. MFI: median fluorescence intensity. Results are expressed as means ± SD (n=3).

**
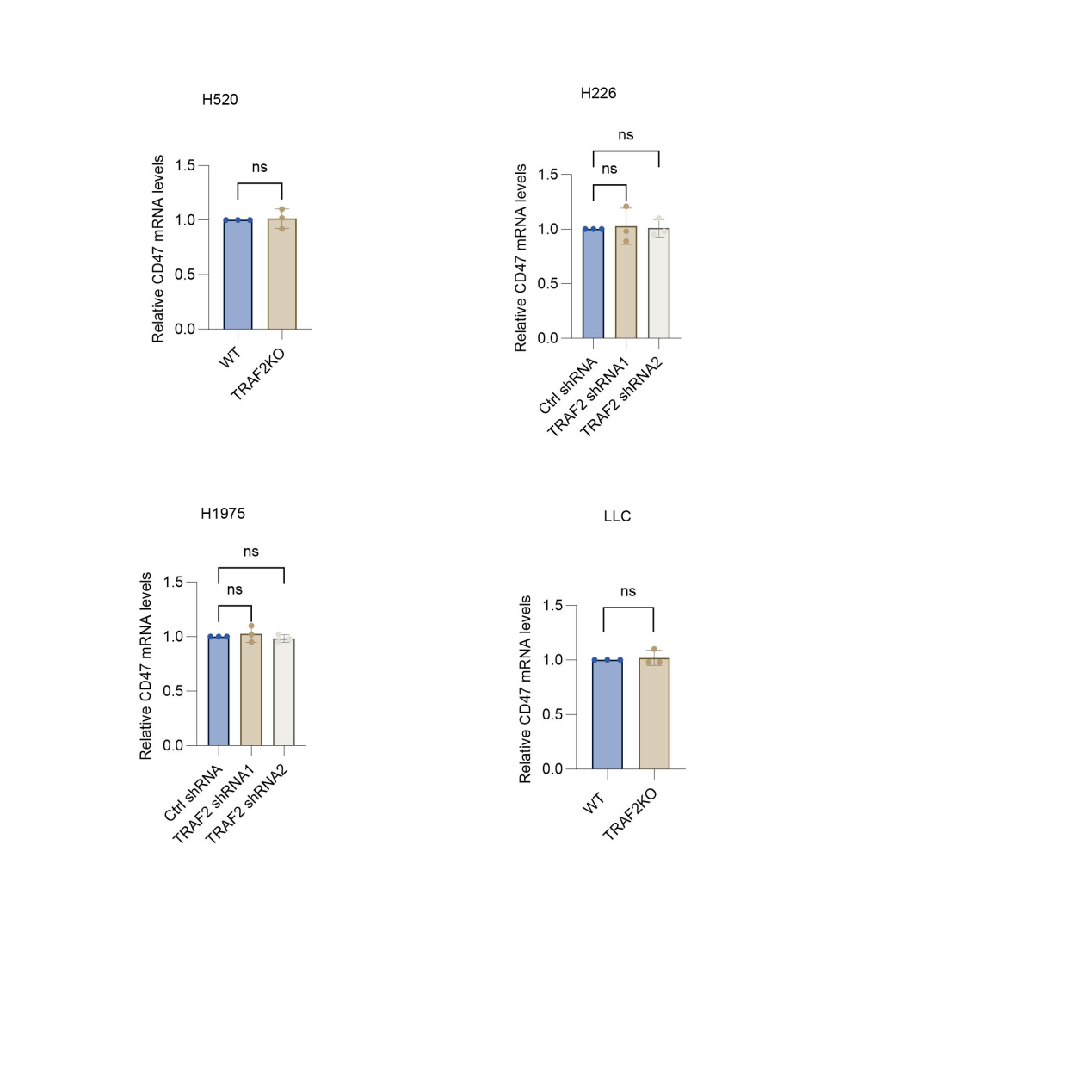
**

**Figure S2. TRAF2 had no effect on CD47 gene levels**

qPCR analysis of CD47 gene expression in WT or TRAF2 gene knockout H520, or LLC cells, and TRAF2 silenced H1975, or H226 cells. Results are expressed as means ± SD (n=3).


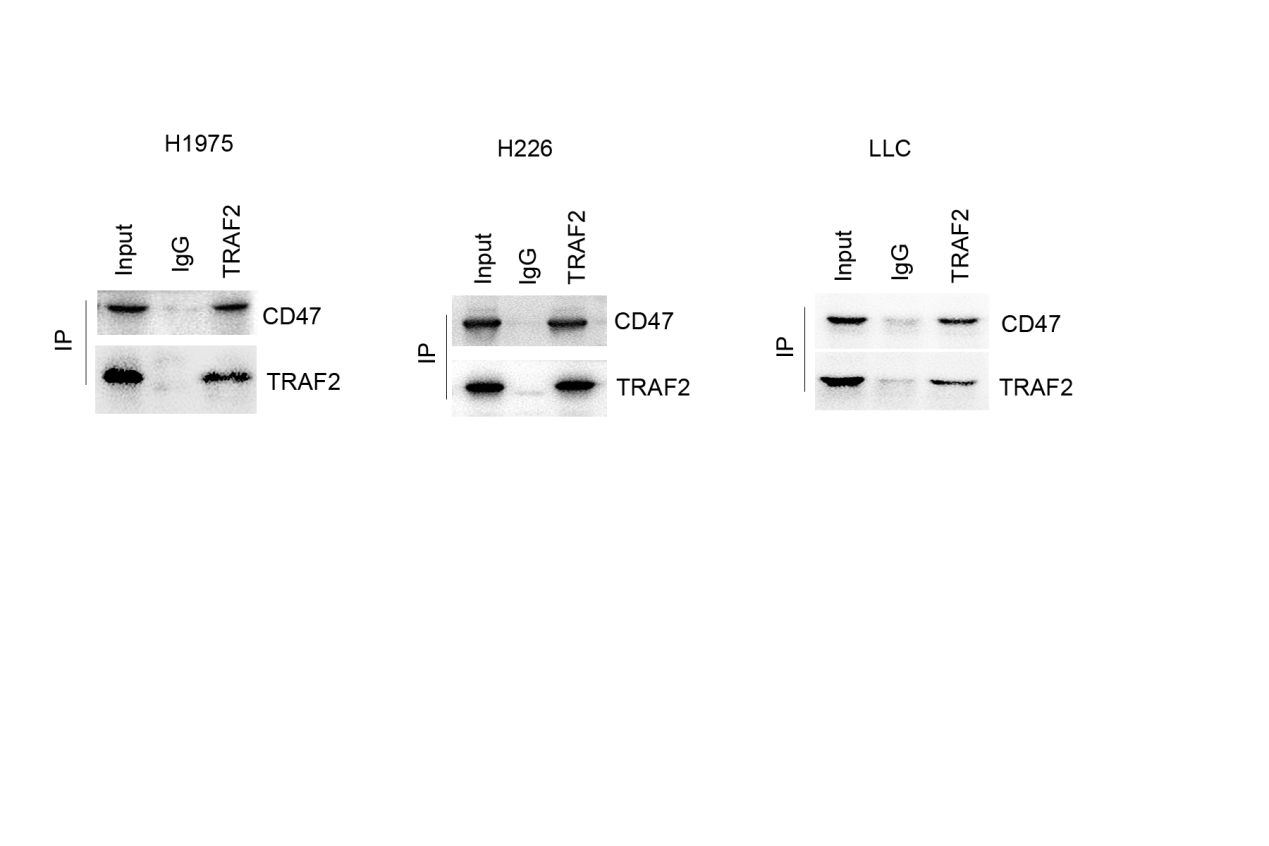


**Figure S3. TRAF2 bound to CD47**

Immunoprecipitation and Western blot analysis of cell lysates

**
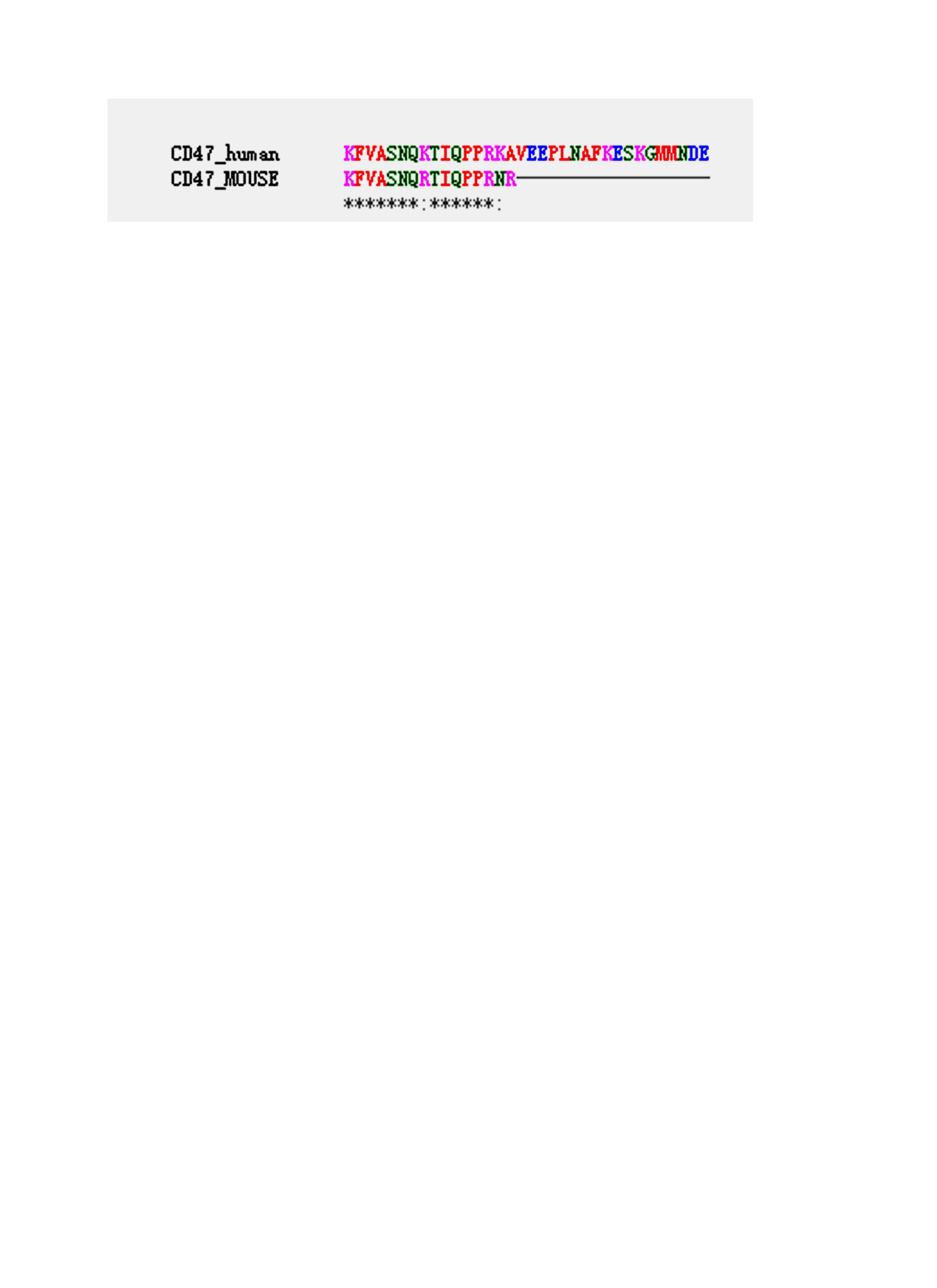
**

**Figure S4. alignment of human and mice CD47 C terminal**

**
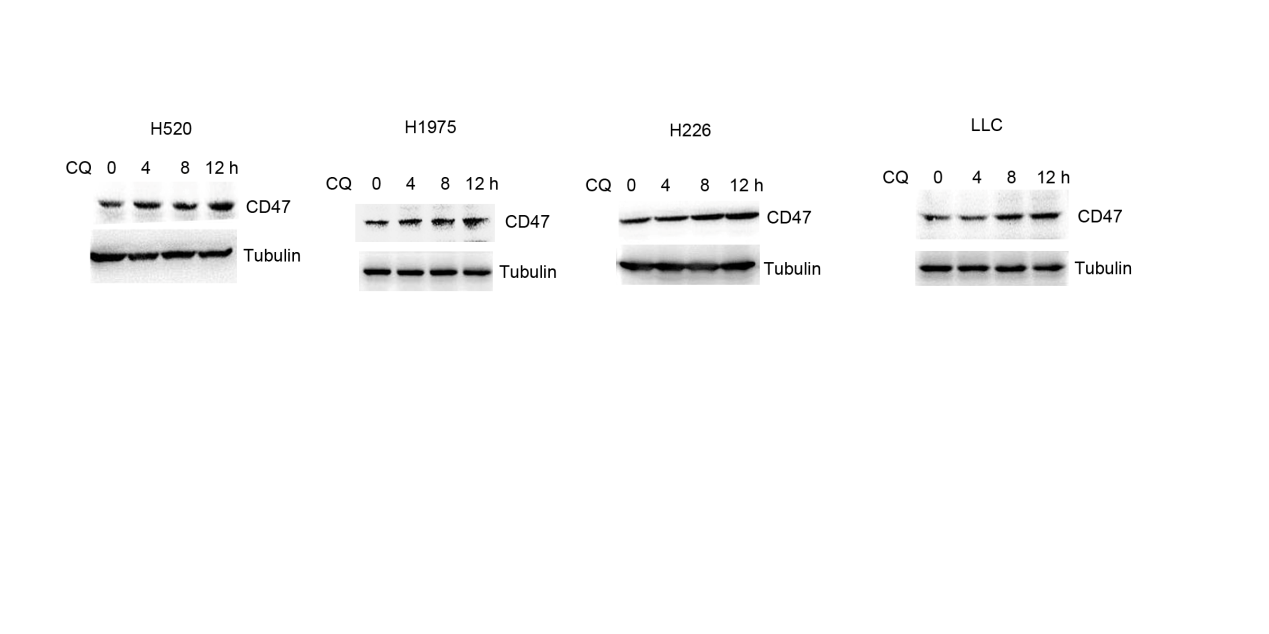
**

**Figure S5. The effect of CD47 levels in response to lysosome inhibitor**

Cells were treated with CQ (30μM) as indicated time course, and cell lysates were subjected to Western blot analysis.

**
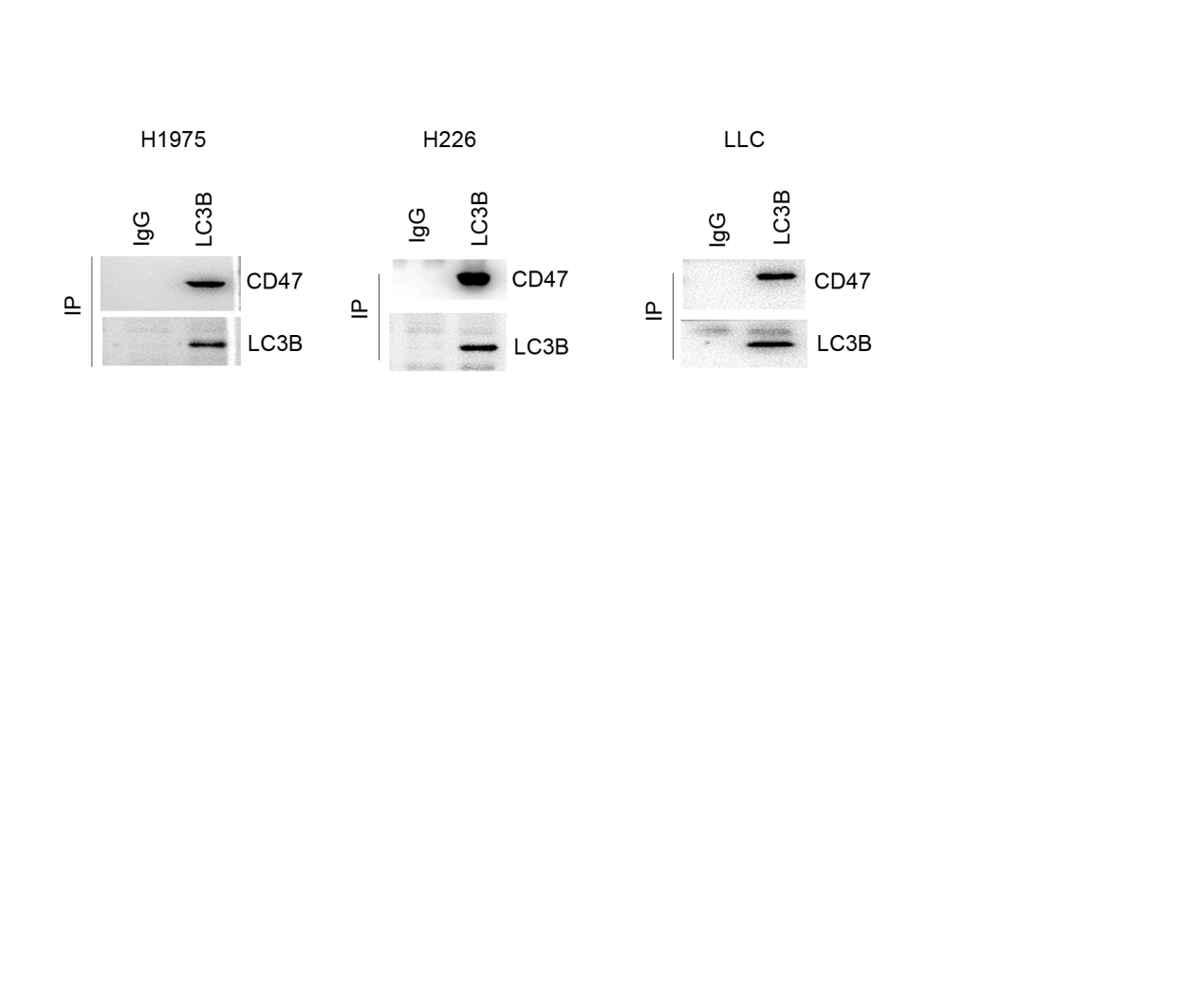
**

**Figure S6. The binding of CD47 to LC3B**

Immunoprecipitation and Western blot analysis of cell lysates

**
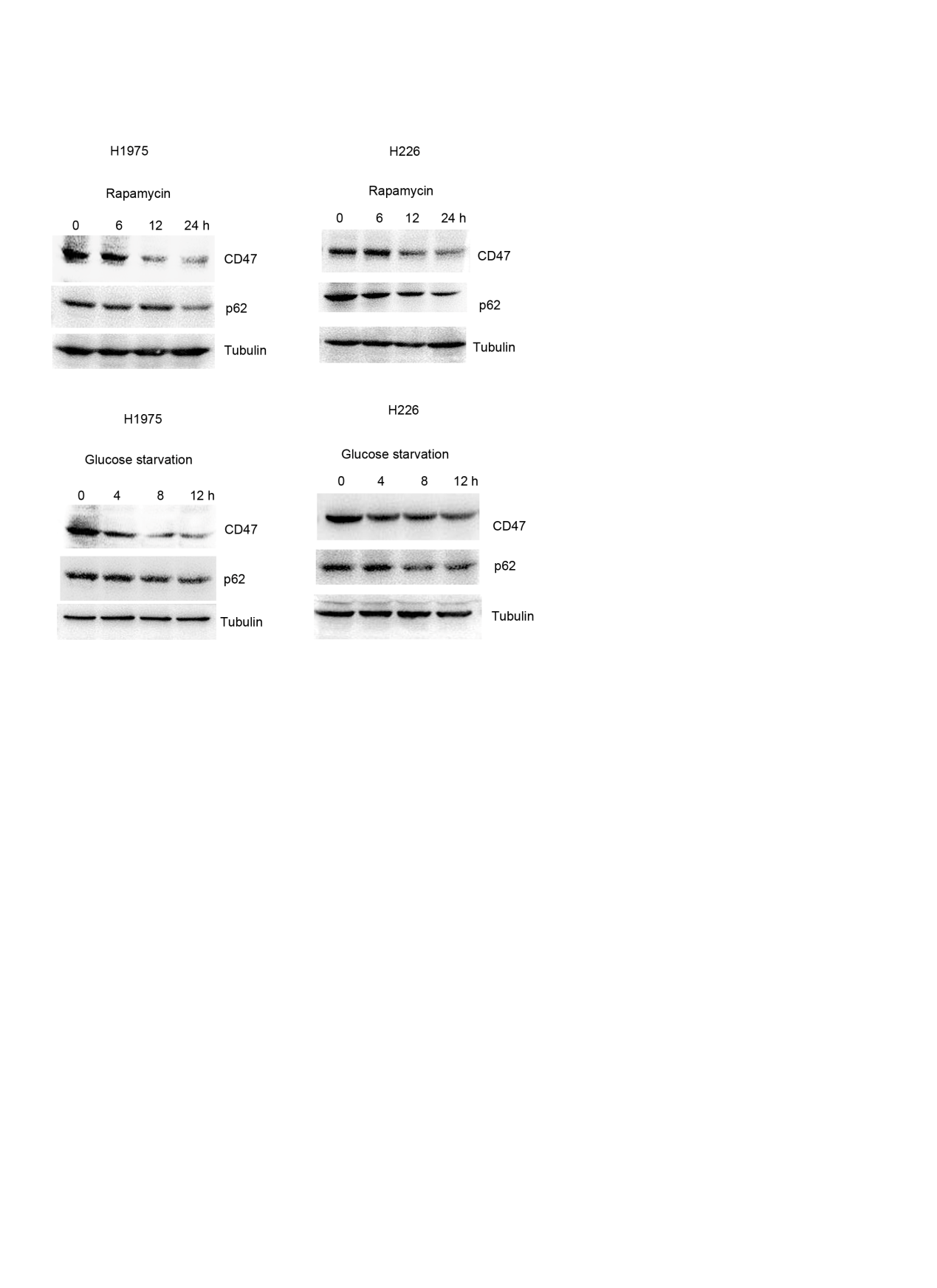
**

**Figure S7. Effect of autophagy induction on CD47 degradation.**

In response to glucose starvation or rapamycin (2μM) treatment, cell lysates were subjected to Western blot analysis.

**
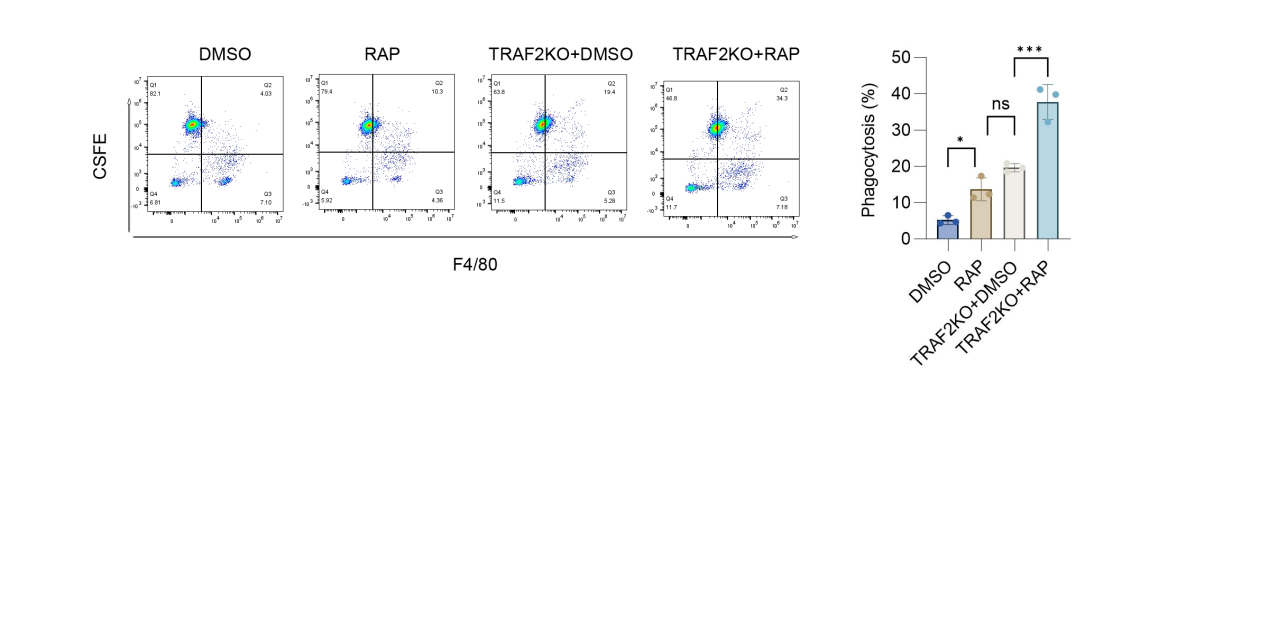
**

**Figure S8. Loss of TRAF2 increased phagocytosis in response to rapamycin**

phagocytosis analysis of WT or TRAF2^-/-^ H520 cells treated without or with rapamycin (2μM) for 12h.

**
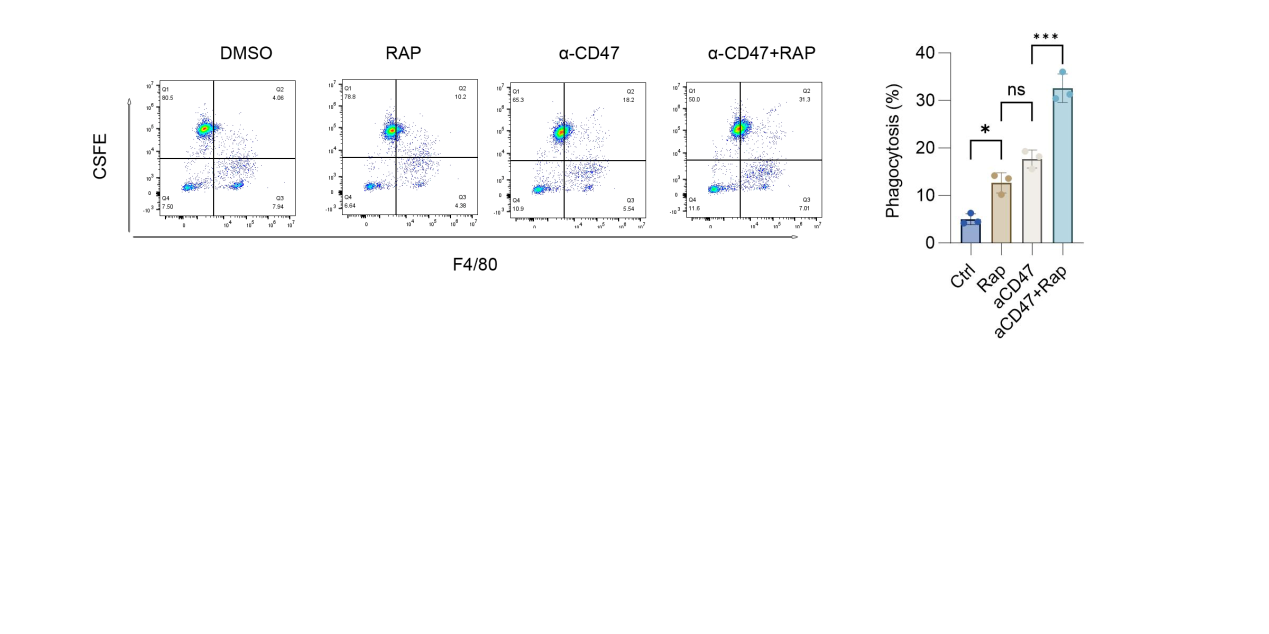
**

**Figure S9. Combined CD47 antibody with rapamycin increased phagocytosis**

phagocytosis analysis of H520 cells treated without or with rapamycin, anti-CD47 mice monoclonal antibody (0.5μg/ml), or rapamycin+anti-CD47 antibody for 12h.
